# Supplementary material for: Supplementary stocking selects for domesticated genotypes
Source: Nat Commun. 2019 Jan 14;10:199. doi: 10.1038/s41467-018-08021-z (PMC6331577; doi:10.1038/s41467-018-08021-z)
Supplement: Supplementary file 1 — Supplementary Information [file 41467_2018_8021_MOESM1_ESM.pdf]

Supplementary stocking selects for domesticated genotypes

Hagen et al.

Supplementary Information

Title: Supplementary stocking selects for domesticated genotypes

Ingerid J. Hagen*[1], Arne J. Jensen[1], Geir H. Bolstad[1], Ola H. Diserud[1], Kjetil Hindar[1], Håvard Lo[2], and Sten Karlsson[1]

1) Norwegian Institute for Nature Research (NINA), P.O. Box 5685 Torgarden, NO-7485 Trondheim, Norway.

2) Norwegian Veterinary Institute, P.O. Box 5695 Torgarden, NO-7485 Trondheim, Norway

**Table 1:** Broodstock information.	Page 2
**Table 2:** Model selection table for regression models with log number of recaptured adult offspring as response.	Page 3
**Table 3.** Parameter estimates for the regression models with egg size as response.	Page 4
**Figure 1.** Effect of introgression (proportion of farmed ancestry) on log egg size (ml) in wild-born and hatchery-reared dams.	Page 5
**Table 4.** Model selection table for regression models with log egg size (ml) as response.	Page 6
**Table 5.** Parameter estimates for the regression models with log number of eggs as response.	Page 7
**Table 6.** Model selection table for regression models with log number of eggs as response.	Page 8
**Table 7.** Parameter estimates for the effect of introgression on log smolt length (mm).	Page 9
**Table 8.** Parameter estimates for the effect of introgression on sea age measured as probability (on logit scale) of maturing given survival to adulthood.	Page 10
**Figure 2.** Effect of introgression (proportion of farmed ancestry) on life history (sea age) in wild spawners returning to River Eira.	Page 11
**Table 9.** Parameter estimates for generalised linear mixed model of proportion farmed ancestry in recaptured wild-born and hatchery-reared adults from 20 run years over a 30-year period.	Page 13
**Table 10.** Number of hatchery-produced Atlantic salmon smolts (out-migrating juveniles) released in River Eira and per cent return to the river estimated as adults per smolt released for cohorts (brood year) 1983 until 2011.	Page 14
**Table 11.** SNP marker information for all markers used.	Page 15
**Supplementary References**	Page 15

Supplementary Table 1 | Broodstock information. Offspring were detected from 77 broodstock dams and 77 broodstock sires with a total of 85 full-sibling groups.

	Broodstock	
	Dam	Sire
Total number individuals	77	77
Individuals used in two crossing	8	8
Hatchery-reared	42	50
Wild-born	35	27
Average proportion farmed ancestry	0.227	0.230

Supplementary Table 2 | Model selection table for regression models with log number of recaptured adult offspring as response. *Int* is the intercept, *#eggs* is the log number of egg, *Eggsz* is the log size of eggs (ml), *ED* is the environmental background (hatchery-reared/wild-born) of broodstock dam, *ES* is the environmental background of broodstock sire, *IPP:ED:ES* is the interaction between introgression shared by broodstock pair and environmental background of the broodstock dam and sire, respectively (hence different effects of introgression shared by the broodstock pair depending on the crosses are wild-born dam × wild-born sire, wild-born dam × hatchery-reared sire, hatchery-reared dam × wild-born sire or hatchery-reared dam × hatchery-reared sire), *IPP:ED* is the interaction between introgression shared by broodstock pair and the environmental background of broodstock dam, *IPP:ES* is the interaction between introgression shared by broodstock pair and the environmental background of the broodstock sire, *df* is the degrees of freedom, *logLik* is the log likelihood, *AICc* is the AICc score, *ΔAIC* is the difference in AICc score compared to best model and *weight* is the AIC weight.

Int	#eggs	Eggsz	ED	ES	IPP:ED:ES	IPP:ED	IPP:ES	IPP	df	logLik	AICc	ΔAIC	weight
-4.587	0.7120		+			+			7	-104.014	223.5	0	0.605
-4.474	0.7078		+	+		+			8	-103.887	225.7	2.19	0.202
-4.230	0.6888		+					0.08399	6	-107.169	227.4	3.93	0.085
-3.507	0.6801	0.3809	+	+		+			9	-103.645	227.7	4.22	0.073
-3.649	0.6786	0.3708	+	+	+				11	-102.219	230.1	6.60	0.022
-4.126	0.6714		+	+			+		8	-106.737	231.4	7.89	0.012
1.522			+			+			6	-113.747	240.6	17.08	0

Supplementary Table 3 | Parameter estimates for the regression models with log egg size (ml) as response. The presented model is the best model. The table gives the parameter estimates ± standard error for each parameter in the model. The effect of hatchery background of dam gives the average difference in number of eggs to wild-born dams. The log covariate weight of dam is mean centred.

Parameter	Best Model Estimates ± SE
Intercept	-1.92607 ± 0.03084
log weight of dam (g)	0.25471 ± 0.04346
Hatchery background of dam	-0.15110 ± 0.03988
Wild-born dam : Introgression	-0.39598 ± 0.14504
Hatchery-reared dam : Introgression	-0.09671 ± 0.05159

Introgression is the proportion of farmed ancestry in mother.

Supplementary Figure 1 | Effect of introgression (proportion of farmed ancestry) on log egg size (ml) in wild-born and hatchery-reared dams. Introgressed wild-born dams produce smaller eggs than wild-born dams with no farmed ancestry. Hatchery-reared dams produce smaller eggs than wild-born dams but show no response to introgression. Lines represent model predictions from least square regression. See Supplementary Table 3 for parameter estimates.

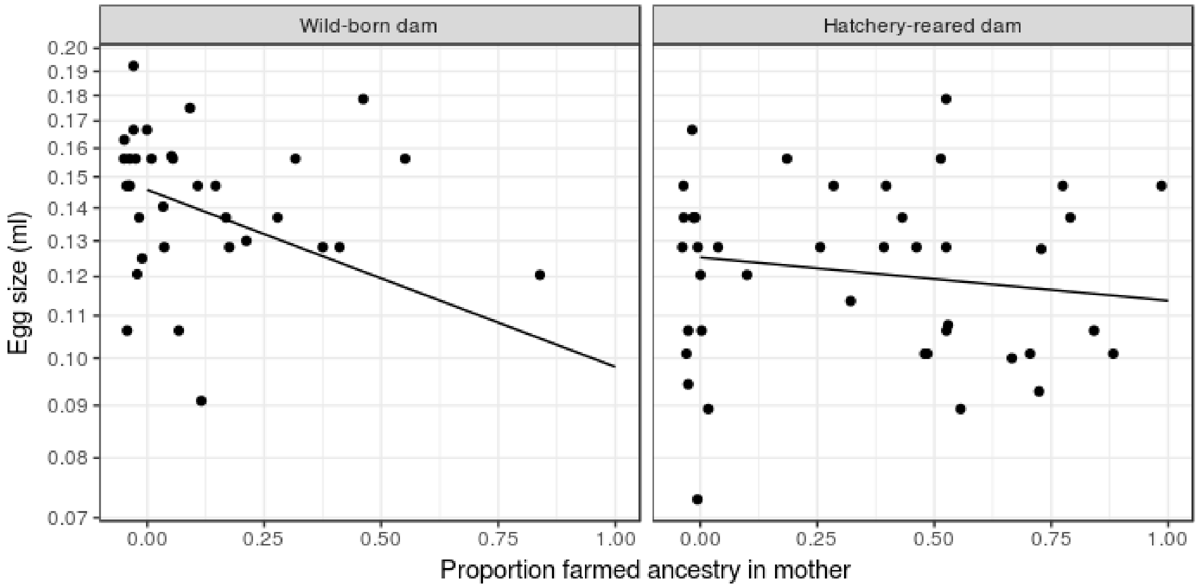

Supplementary Table 4 | Model selection table for regression models with log egg size (ml) as response. *Int* is the intercept, *log(weight)* is the mean centred log weight of the broodstock dam (g), *ID* is the introgression (proportion of farmed ancestry) in the dam, *HD* is the hatchery background of broodstock dam, *ID:HD* is the interaction between broodstock dam introgression and hatchery background, *df* is the degrees of freedom, *LogLik* is the log likelihood, *AICc* is the AICc score, *ΔAIC* is the difference in AICc score compared to best model and *weight* is the AIC weight.

Int	log(weight)	ID	HD	ID:HD	df	LogLik	AICc	ΔAIC	weight
-1.924	0.2547	-0.3963	+	+	7	41.552	-67.5	0	0.625
-1.936	0.2521	-0.1427	+		6	39.731	-66.2	1.21	0.341
-1.951	0.2322		+		5	36.252	-61.6	5.81	0.034
-1.931		-0.3250	+	+	6	26.622	-40.0	27.43	0.000

Supplementary Table 5 | Parameter estimates for the regression models with log number of eggs as response. Each column gives the parameter estimates ± standard error for each parameter in the three models. The effect of hatchery background of dam gives the average difference in number of eggs to wild-born dams. The covariate log weight of dam is mean centred.

Parameter	Best model Estimates ± SE	Best model + dam introgression Estimates ± SE
Intercept	8.75128 ± 0.06236	8.74709 ± 0.06180
log weight of dam (g)	0.74275 ± 0.09817	0.73378 ± 0.10006
Hatchery background of dam	0.17615 ± 0.07808	0.16312 ± 0.08477
Dam introgression		0.05365 ± 0.11106
ΔAIC	0.0	1.98

Introgression is the proportion of farm ancestry in the dam.

Supplementary Table 6 | Model selection table for regression models with log number of eggs as response. *Int* is the intercept, *Eggsz* is the log egg size, *log(weight)* is the mean centred log weight (g) of the broodstock dam, *ID* is the introgression proportion of farmed ancestry in the broodstock dam, *HD* is the hatchery background of the broodstock dam, *ID:HD* is the interaction between dam broodstock introgression and hatchery background of the broodstock dam, *df* is the degrees of freedom, *logLik* is the log likelihood, *AICc* is the AICc score, *ΔAIC* is the difference in AICc score compared to the best model and *weight* is the AIC weight.

Int	Eggsz	log(weight)	ID	HD	ID:HD	df	logLik	AICc	ΔAIC	weight
8.748		0.7387		+		5	-22.272	55.4	0	0.448
8.742		0.7276	0.06729	+		6	-22.081	57.4	1.98	0.167
8.769		0.7311	-0.3454	+	+	7	-21.197	58	2.64	0.12
8.848		0.7553				4	-24.803	58.2	2.77	0.112
8.81	-0.3351	0.8163	-0.4781	+	+	8	-20.365	58.9	3.48	0.079
8.82		0.7324	0.1319			5	-24.08	59	3.61	0.074

Supplementary Table 7 | Parameter estimates for the effect of introgression on log smolt length (mm). The intercepts are given by *a*, where the subscript denotes sea age in years, the effect of level of introgression (proportion of farmed ancestry of the smolt) is given by *b*. *N* gives the sample size.

	Hatchery-reared		Wild-born	
Parameter	Estimate	Standard error	Estimate	Standard error
a_1	5.348	0.027	4.909	0.014
a_2	5.375	0.027	4.928	0.015
a_3	5.411	0.031	4.970	0.024
a_4	5.351	0.050	4.893	0.074
b	0.060	0.019	0.055	0.030
N	892		966	

Supplementary Table 8 | Parameter estimates for the effect of introgression on sea age measured as probability (on logit scale) of maturing given survival to adulthood. The intercept is given by *a*, where the subscript denotes sea age in years. The within year effect of level of introgression (proportion of farmed ancestry of each fish) is given by *b*, and the difference between the within- and among-year effect of level of introgression is given by *d*. *N* gives the sample size.

		Hatchery-reared		Wild-born	
Sex	Parameter	Estimate	Standard error	Estimate	Standard error
Female	a_1	0.34	0.54	1.32	0.40
	a_2	0.49	0.47	0.98	0.36
	b_1	0.47	0.36	-0.47	0.54
	b_2	0.32	0.31	0.12	0.40
	d_1	0.14	1.65	-8.95	3.24
	d_2	0.94	1.48	0.99	2.78
	N	545		580	
Male	a_1	2.01	0.73	1.27	0.40
	a_2	0.99	0.63	0.19	0.38
	b_1	0.18	0.32	0.93	0.72
	b_2	-0.03	0.32	0.82	0.71
	d_1	-2.52	2.50	4.60	3.83
	d_2	-0.02	2.12	10.83	3.69
	N	751		584	

Supplementary Figure 2 | Effect of introgression (proportion of farmed ancestry) on life history (sea age) in wild spawners returning to River Eira. (a) Effect of introgression on sea age at maturation (proportion of each sea-age category: 1, 2 and ≥ 3 years) for hatchery-reared females and males. **(b)** Effect of introgression on sea age at maturation (proportion of each sea-age category: 1, 2 and ≥ 3 years) for wild-born females and males. Lines represent model predictions and shaded areas represent the standard error (symmetrical on the logit scale). Statistical support is given by the difference in the Akaike information criterion (ΔAIC) between a model excluding the effect of introgression and one including it. A Δ AIC value more negative than − 2 is considered strong statistical support. See Supplementary Table 8 for parameter estimates.

a

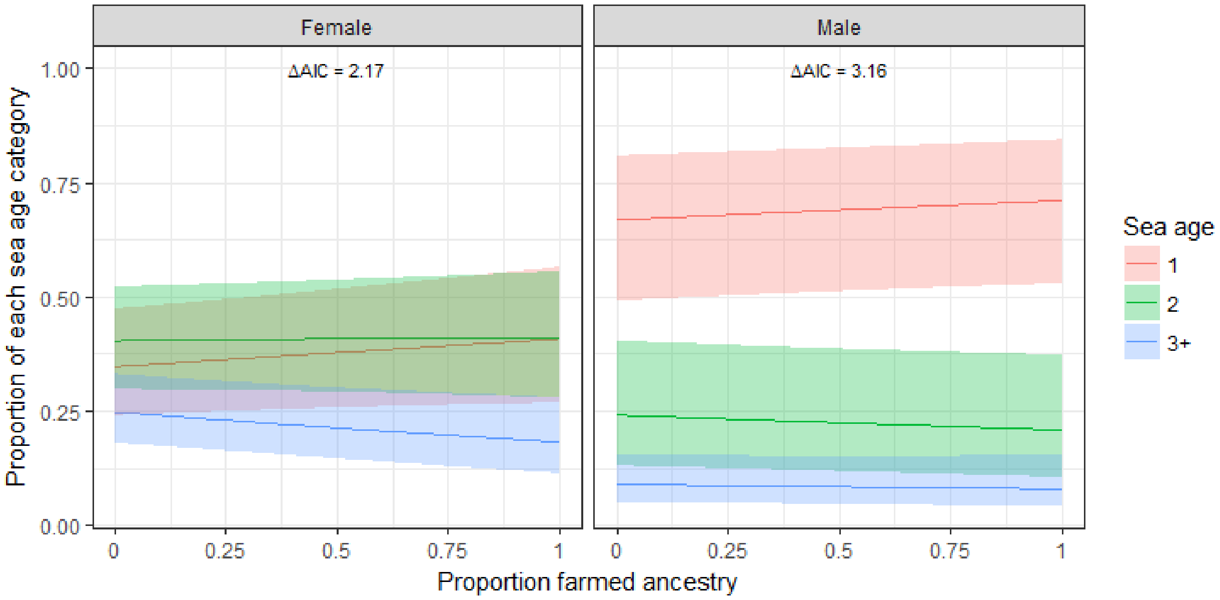

b

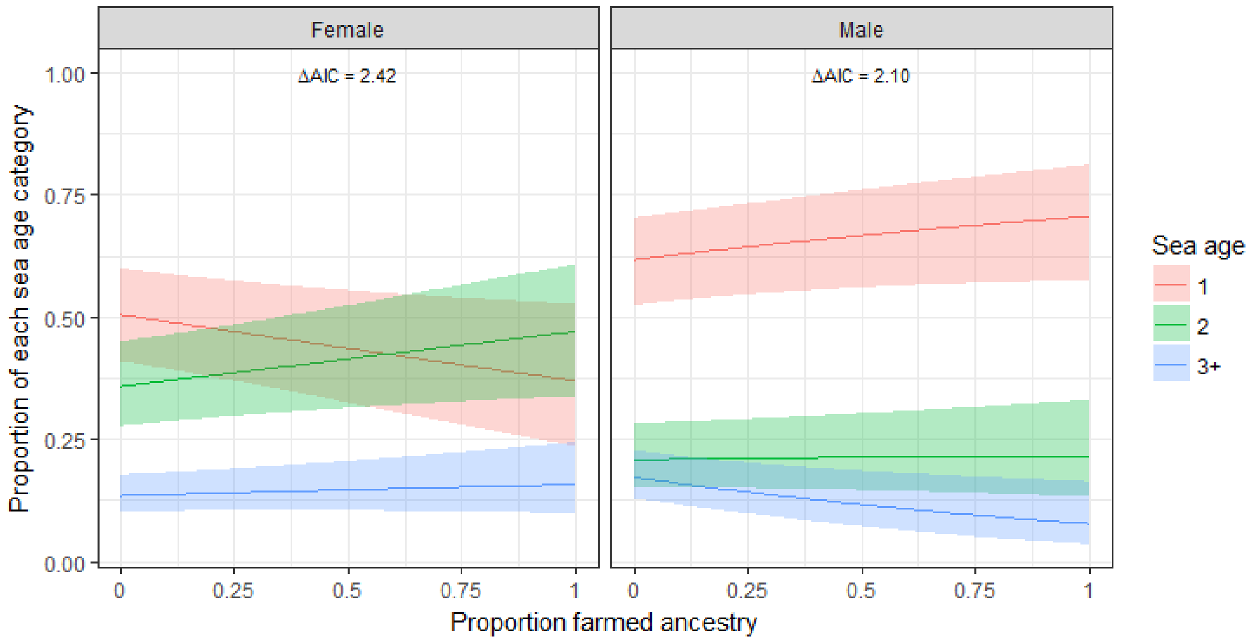
Female
Male
ΔAIC = 2.42
ΔAIC = 2.10
Proportion of each sea age category
1.00
0.75
0.50
0.25
0.00
0
0.25
0.5
0.75
1
Proportion farmed ancestry
Sea age
1
2
3+

Supplementary Table 9 | Parameter estimates for generalised linear model of proportion farmed ancestry in recaptured wild-born and hatchery-reared adults from 20 run years over a 30-year period. Each row gives the sample size, difference in logit proportion farmed ancestry (Δ) and the standard error between hatchery-reared and wild-born adult spawners for each run year.

	Sample size		Difference in proportional domesticated ancestry		
Run year	Hatchery	Wild	Δ	Standard error	P-value
1987	12	46	0.048	0.609	0.94
1988	7	28	-1.070	0.792	0.17
1989	21	59	-0.599	0.477	0.21
1990	24	65	-0.976	0.448	0.03 *
1991	7	24	-1.296	0.805	0.11
1992	8	20	-1.005	0.784	0.20
1998	18	13	-2.403	0.682	0.0004 ***
2000	43	34	-1.134	0.430	0.008 **
2001	26	65	-0.687	0.435	0.11
2006	52	17	-1.046	0.524	0.046*
2007	99	46	-0.977	0.334	0.0035 **
2008	149	68	-0.612	0.274	0.026 *
2009	93	122	-1.193	0.258	< 0.0001 ***
2010	165	154	-1.510	0.210	< 0.0001 ***
2011	245	133	-1.061	0.202	< 0.0001 ***
2012	188	81	-0.740	0.249	0.003 **
2013	91	54	-0.230	0.322	0.47
2014	66	125	-0.844	0.285	0.003**
2015	151	109	-1.200	0.236	< 0.0001 ***
2016	102	84	-0.828	0.276	0.003 **
Total	1567	1347			

Supplementary Table 10 | Number of hatchery-produced Atlantic salmon smolts (out-migrating juveniles) released in River Eira and per cent return to the river estimated as adults per smolt released for cohorts (brood year) 1983 until 2011. Overall adult return rates per cohort were estimated by combining data on rod catches, individuals used for broodstock, and counts of the spawning population (assessed by snorkelling surveys)[1].

Brood year	Released smolts 1 / 2-year-old	Return rate (%)
1983	0 / ca. 50 000	-
1988	0 / ca. 50 000	-
1989	0 / 58 651	0.00*
1990	0 / 57 110	0.16*
1991	0 / 50 139	0.08*
1992	0 / 52 891	0.00*
1998	0 / 50 981	0.44
2000	0 / 54 224	0.61
2005	0 / 51 415	0.66
2006	0 / 63 000	1.03
2007	0 / 57 000	0.68
2008	0 / 60 000	0.90
2009	12 000 / 57 000	0.15 / 0.17
2010	8 000 / 34 000	0.57 / 1.05
2011	39 800 / 35 400	0.00 / 1.49

*Recapture rate of Carlin-tagged smolts

Supplementary Table 11 | SNP marker information for all markers used. SNP id, type (nuclear or mitochondrial), use (for estimating farmed genetic introgression (P(wild)), parentage assignment, and match of haplotype between mother and offspring) and ss# in dbSNP or accession # in Genbank.

SNP id	Type	Use	ss# in dbSNP or accession # in Genbank
BASS111_B7_B11_707	Nuclear	P(wild) and parentage assignment	ss130452355
ESTNV_28129_298	Nuclear	P(wild) and parentage assignment	ss262994526
ESTNV_18603_371	Nuclear	P(wild) and parentage assignment	ss262976736
ESTNV_31226_313	Nuclear	P(wild) and parentage assignment	ss262995082
GCR_cBin14283_Ctg1_117	Nuclear	P(wild) and parentage assignment	ss262981858
ESTNV_36692_1354	Nuclear	P(wild) and parentage assignment	ss262996838
GCR_cBin40148_Ctg1_35	Nuclear	P(wild) and parentage assignment	ss262990174
GCR_cBin10025_Ctg1_195	Nuclear	P(wild) and parentage assignment	ss262981465
ESTNV_14268_378	Nuclear	P(wild) and parentage assignment	ss262985303
ESTNV_28225_649	Nuclear	P(wild) and parentage assignment	ss263002743
ESTNV_23346_206	Nuclear	P(wild) and parentage assignment	ss262994065
ESTNV_32346_630	Nuclear	P(wild) and parentage assignment	ss263003271
ESTV_19099_538	Nuclear	P(wild) and parentage assignment	ss262998067
ESTNV_19288_484	Nuclear	P(wild) and parentage assignment	ss263002266
ESTNV_35257_1292	Nuclear	P(wild) and parentage assignment	ss262996273
ESTNV_34021_1870	Nuclear	P(wild) and parentage assignment	ss262995848
GCR_cBin32565_Ctg1_59	Nuclear	P(wild) and parentage assignment	ss262989868
GCR_cBin23727_Ctg1_154	Nuclear	P(wild) and parentage assignment	ss262982624
GCR_cBin15671_Ctg1_125	Nuclear	P(wild) and parentage assignment	ss262998859
ESTNV_32013_264	Nuclear	P(wild) and parentage assignment	ss262995261
ESTNV_29115_481	Nuclear	P(wild) and parentage assignment	ss262977529
GCR_cBin24261_Ctg1_69	Nuclear	P(wild) and parentage assignment	ss262989424
GCR_cBin2854_Ctg1_228	Nuclear	P(wild) and parentage assignment	ss262989654

BASS119_B7_E10_373	Nuclear	P(wild) and parentage assignment	ss130452381
ESTNV_32170_318	Nuclear	P(wild) and parentage assignment	ss262995296
ESTNV_35759_1059	Nuclear	P(wild) and parentage assignment	ss262996442
ESTNV_31647_643	Nuclear	P(wild) and parentage assignment	ss262995167
GCR_cBin10148_Ctg1_91	Nuclear	P(wild) and parentage assignment	ss262988584
ESTNV_35893_841	Nuclear	P(wild) and parentage assignment	ss263004043
GCR_cBin21600_Ctg1_99	Nuclear	P(wild) and parentage assignment	ss262982454
ESTNV_33766_718	Nuclear	P(wild) and parentage assignment	ss262995758
ESTNV_31163_234	Nuclear	P(wild) and parentage assignment	ss263003101
GCR_cBin17883_Ctg1_169	Nuclear	P(wild) and parentage assignment	ss262982172
GCR_cBin4844_Ctg1_156	Nuclear	P(wild) and parentage assignment	ss262984273
BASS123_B7_B04_360	Nuclear	P(wild) and parentage assignment	ss130452392
ESTNV_34419_496	Nuclear	P(wild) and parentage assignment	ss262995980
ESTNV_36086_719	Nuclear	P(wild) and parentage assignment	ss262996577
ESTV_15230_258	Nuclear	P(wild) and parentage assignment	ss262997515
GCR_cBin1955_Ctg1_60	Nuclear	P(wild) and parentage assignment	ss262982310
GCR_cBin34264_Ctg1_201	Nuclear	P(wild) and parentage assignment	ss262989947
GCR_cBin5182_Ctg1_123	Nuclear	P(wild) and parentage assignment	ss262984427
ESTNV_34639_1192	Nuclear	P(wild) and parentage assignment	ss263003725
ESTNV_36576_1908	Nuclear	P(wild) and parentage assignment	ss262996812
ESTNV_31902_575	Nuclear	P(wild) and parentage assignment	ss262995234
GCR_cBin13217_Ctg1_178	Nuclear	P(wild) and parentage assignment	ss262981773
GCR_cBin21396_Ctg1_111	Nuclear	P(wild) and parentage assignment	ss262982434
GCR_cBin49912_Ctg1_98	Nuclear	P(wild) and parentage assignment	ss262990485
MHC_IA_33360-33530	Nuclear	P(wild) and parentage assignment	ss263198250
ESTV_15868_527	Nuclear	Parentage assignment	ss263004846
ESTV_17429_1139	Nuclear	Parentage assignment	ss262997980
ESTV_11898_329	Nuclear	Parentage assignment	ss262980209
ESTV_17454_240	Nuclear	Parentage assignment	ss262997988

GCR_cBin15343_Ctg1_36	Nuclear	Parentage assignment	ss262981960
GCR_cBin22215_Ctg1_184	Nuclear	Parentage assignment	ss262982513
GCR_cBin6795_Ctg1_308	Nuclear	Parentage assignment	ss262984647
ESTNV_28793_1230	Nuclear	Parentage assignment	ss262994610
ESTV_13454_78	Nuclear	Parentage assignment	ss263004603
GCR_cBin17226_Ctg1_170	Nuclear	Parentage assignment	ss262989016
GCR_cBin42428_Ctg1_28	Nuclear	Parentage assignment	ss262983963
ESTNV_22611_642	Nuclear	Parentage assignment	ss263002367
ESTV_14201_395	Nuclear	Parentage assignment	ss262997371
ESTV_17015_1763	Nuclear	Parentage assignment	ss263005023
ESTV_17611_91	Nuclear	Parentage assignment	ss262998004
GCR_cBin17287_Ctg1_341	Nuclear	Parentage assignment	ss262989020
GCR_cBin47268_Ctg1_70	Nuclear	Parentage assignment	ss262990421
GCR_hBin32129_Ctg1_119	Nuclear	Parentage assignment	ss262990971
ESTV_17067_557	Nuclear	Parentage assignment	ss262997904
GCR_cBin17484_Ctg1_144	Nuclear	Parentage assignment	ss262989040
GCR_cBin48270_Ctg1_181	Nuclear	Parentage assignment	ss262984260
ESTNV_23996_351	Nuclear	Parentage assignment	ss262985608
ESTV_14711_157	Nuclear	Parentage assignment	ss262997436
ESTV_17112_405	Nuclear	Parentage assignment	ss262997918
GCR_cBin10938_Ctg1_42	Nuclear	Parentage assignment	ss262981538
GCR_cBin3299_Ctg1_307	Nuclear	Parentage assignment	ss262983381
GCR_rBin4036_Ctg1_280	Nuclear	Parentage assignment	ss262985250
ESTV_17222_301	Nuclear	Parentage assignment	ss262997938
GCR_cBin11008_Ctg1_366	Nuclear	Parentage assignment	ss262988632
ESTNV_26881_400	Nuclear	Parentage assignment	ss262994352
ESTV_17428_1391	Nuclear	Parentage assignment	ss263005065
GCR_cBin40_Ctg1_186	Nuclear	Parentage assignment	ss262983822
GCR_cBin5337_Ctg1_532	Nuclear	Parentage assignment	ss262990572
Mito_ND1_3989	Mitochondrial	Mother-offspring match	EU643670-EU643674
Mito_ND4_11937	Mitochondrial	Mother-offspring match	EU643682-EU643686
Mito_ND1_4517	Mitochondrial	Mother-offspring match	EU643670-EU643674
Mito_ND4_12260	Mitochondrial	Mother-offspring match	EU643682-EU643686
Mito_ND2_5365	Mitochondrial	Mother-offspring match	EU643675-EU643679
Mito_ND5_13479	Mitochondrial	Mother-offspring match	EU643687-EU643690
Mito_ND5_13536	Mitochondrial	Mother-offspring match	EU643687-EU643690
Mito_ND2_5768	Mitochondrial	Mother-offspring match	EU643675-EU643679
Mito_ND5_14017	Mitochondrial	Mother-offspring match	EU643687-EU643690
Mito_ND3_10879	Mitochondrial	Mother-offspring match	EU643680-EU643681
Mito_ND5_14111	Mitochondrial	Mother-offspring match	EU643687-EU643690

Mito_D-loop_703	Mitochondrial	Mother-offspring match	ss263007721
Mito_ND4_11495	Mitochondrial	Mother-offspring match	EU643682-EU643686
Mito_ND6_14895	Mitochondrial	Mother-offspring match	EU643691-EU643692
Mito_ND1_3900	Mitochondrial	Mother-offspring match	EU643670-EU643674

Supplementary References

1) Jensen AJ, Berg M, Bremset G, Finstad B, Havn TB, Jensås JG. Fiskebiologiske undersøkelser i Auravassdraget. Årsrapport for 2015. NINA Rapport 1249. Norwegian Institute for Nature Research (2016).
