# Supplementary material for: Supplementary stocking selects for domesticated genotypes
Source: Nat Commun. 2019 Jan 14;10:199. doi: 10.1038/s41467-018-08021-z (PMC6331577; doi:10.1038/s41467-018-08021-z)
Supplement: Supplementary file 3 — Reporting Summary [file 41467_2018_8021_MOESM3_ESM.pdf]

nature research

Corresponding author(s): Ingerid Julie Hagen
ingerid.arnesen@nina.no

Reporting Summary

Nature Research wishes to improve the reproducibility of the work that we publish. This form provides structure for consistency and transparency in reporting. For further information on Nature Research policies, see Authors & Referees and the Editorial Policy Checklist.

Statistical parameters

When statistical analyses are reported, confirm that the following items are present in the relevant location (e.g. figure legend, table legend, main text, or Methods section).

n/a	Confirmed	
☐	☒	The exact sample size (*n*) for each experimental group/condition, given as a discrete number and unit of measurement
☐	☒	An indication of whether measurements were taken from distinct samples or whether the same sample was measured repeatedly
☐	☒	The statistical test(s) used AND whether they are one- or two-sided<br>*Only common tests should be described solely by name; describe more complex techniques in the Methods section.*
☐	☒	A description of all covariates tested
☐	☒	A description of any assumptions or corrections, such as tests of normality and adjustment for multiple comparisons
☐	☒	A full description of the statistics including central tendency (e.g. means) or other basic estimates (e.g. regression coefficient) AND variation (e.g. standard deviation) or associated estimates of uncertainty (e.g. confidence intervals)
☐	☒	For null hypothesis testing, the test statistic (e.g. *F*, *t*, *r*) with confidence intervals, effect sizes, degrees of freedom and *P* value noted<br>*Give P values as exact values whenever suitable.*
☐	☒	For Bayesian analysis, information on the choice of priors and Markov chain Monte Carlo settings
☐	☒	For hierarchical and complex designs, identification of the appropriate level for tests and full reporting of outcomes
☐	☒	Estimates of effect sizes (e.g. Cohen's *d*, Pearson's *r*), indicating how they were calculated
☐	☒	Clearly defined error bars<br>*State explicitly what error bars represent (e.g. SD, SE, CI)*

Our web collection on statistics for biologists may be useful.

Software and code

Policy information about availability of computer code

Data collection	No software was used to collect the data.
Data analysis	The data in this study were analyzed using custom code in R.

For manuscripts utilizing custom algorithms or software that are central to the research but not yet described in published literature, software must be made available to editors/reviewers upon request. We strongly encourage code deposition in a community repository (e.g. GitHub). See the Nature Research guidelines for submitting code & software for further information.

Data

Policy information about availability of data

All manuscripts must include a data availability statement. This statement should provide the following information, where applicable:

- Accession codes, unique identifiers, or web links for publicly available datasets
- A list of figures that have associated raw data
- A description of any restrictions on data availability

The data supporting the findings of this study are available in the Dryad Digital Repository with the identifier doi:10.5061/dryad.1nh877d. All other relevant data is available upon request.

Field-specific reporting

Please select the best fit for your research. If you are not sure, read the appropriate sections before making your selection.

☐ Life sciences ☐ Behavioural & social sciences ☒ Ecological, evolutionary & environmental sciences

For a reference copy of the document with all sections, see nature.com/authors/policies/ReportingSummary-flat.pdf

Ecological, evolutionary & environmental sciences study design

All studies must disclose on these points even when the disclosure is negative.

Study description	We related reproductive success of broodfish to introgression with farmed escapees in an Atlantic salmon stocking program. We also compared farm introgression between hathcery-reared and wild-born fish in the recipient population.
Research sample	The research sample consists of adult spawners of Atlantic salmon (Salmo salar) returning to River Eira. The fish were either caught and killed by fishermen during the angling season, or were caught and used as broodfish in the River Eira stocking program. Scale samples were submitted voluntarily to the Norwegian Veterinary Institute and the Norwegian Institute for Nature Research. Genotyping of these samples were done to assess the effect of the River Eira stocking program. This study represent an important part of the assessment.
Sampling strategy	No sample-size calculation has been done. We used already collected data and defined hypotheses that were tested using the available data.
Data collection	Scale samples were submitted voluntarily by sport anglers. The scales were further analyzed in the lab, by genotyping and reading of the scales.
Timing and spatial scale	Broodstock used in the brood years 2005-2011 were genotyped. Samples from sports fishing that were assigned to the brood years 1983-1986, 1993-1996 and 2002-2011 were genotyped. The selection was done to determine reproductive success of the genotyped broodfish, and to assess the effect of stocking in River Eira. These samples correspond to run years (catch years) between 1987 - 2016.
Data exclusions	We used all available genotyped samples. Some run years (catch years) did not have samples representing both groups, and a comparison between the groups could not be done. As such there are gaps in the 1987 - 2016 data.
Reproducibility	No experiment has been performed to obtain the data reported in this study. We have used data collected from nature and from the River Eira stocking program.
Randomization	No randomization was required. We related reproductive success to introgression and compared introgression between two groups: hatchery-released and wild-born. These groups cannot be randomized.
Blinding	Blinding was not relevant in this study. We related introgression in broodfish to the number of recaptured offspring and compared introgresion in two groups of individuals.

Did the study involve field work? ☐ Yes ☒ No

Reporting for specific materials, systems and methods

Materials & experimental systems

n/a	Involved in the study
☒	☐ Unique biological materials
☒	☐ Antibodies
☒	☐ Eukaryotic cell lines
☒	☐ Palaeontology
☐	☒ Animals and other organisms
☒	☐ Human research participants

Methods

n/a	Involved in the study
☒	☐ ChIP-seq
☒	☐ Flow cytometry
☒	☐ MRI-based neuroimaging

April 2018

Animals and other organisms

Policy information about studies involving animals; ARRIVE guidelines recommended for reporting animal research

Laboratory animals	The study did not involve laboratory animals.
Wild animals	The study involved 1) use of scale samples that were submitted voluntarily by sport anglers upon the legal capture of a fish during the summer angling season, and 2) scale samples collected from broodfish used in the stocking program in River Eira.
Field-collected samples	The study used scale samples collected form wild animals as described under 'Wild animals' above.
